# Phenotyping progression of secondary mitral regurgitation in chronic systolic heart failure

**DOI:** 10.1111/eci.13159

**Published:** 2019-10-09

**Authors:** Henrike Arfsten, Philipp E Bartko, Noemi Pavo, Gregor Heitzinger, Julia Mascherbauer, Christian Hengstenberg, Martin Hülsmann, Georg Goliasch

**Affiliations:** ^1^ Department of Internal Medicine II, Division of Cardiology Medical University of Vienna Vienna Austria

**Keywords:** HFrEF, neurohumoral marker, sMR, sMR progression

## Abstract

**Background:**

Secondary mitral regurgitation (sMR) drives adverse cardiac remodelling in patients with heart failure with reduced ejection fraction (HFrEF). Progression in severity over time contributes to a transition towards more advanced HF stages. Early identification of patients at risk for sMR progression remains challenging. We therefore sought to assess a broad spectrum of neurohumoral biomarkers in patients with HFrEF to explore their ability to predict progression of sMR.

**Methods:**

A total of 249 HFrEF patients were enrolled. Biomarkers encompassing key neurohumoral pathways in heart failure were sampled at baseline, and sMR progression was assessed over 3 years of follow‐up.

**Results:**

Of 191 patients with nonsevere sMR at baseline, 18% showed progressive sMR within three years after study enrolment. Progression of sMR was associated with higher levels of MR‐proADM (adj.OR 2.25, 95% CI 1.29‐3.93; *P* = .004), MR‐proANP (adj.OR 1.84, 95% CI 1.14‐3.00; *P* = .012), copeptin (adj.OR 1.66, 95% CI 1.04‐2.67; *P* = .035) and CT‐pro‐ET1 (adj.OR 1.68, 95% CI 1.06‐2.68; *P* = .027) but not with NT‐proBNP (*P* = .54).

**Conclusion:**

Increased plasma levels of neurohumoral cardiac biomarkers are predictors of sMR progression in patients with HFrEF and add easily available incremental prognostic information for risk stratification. Importantly, NT‐proBNP was not useful to predict progressive sMR in the present analysis. On the contrary, MR‐proANP, primarily produced in the atria, copeptin partly triggered by intra‐cardiac and intra‐arterial pressures and MR‐proADM, a marker of forward failure and peripheral released vasoactive CT‐proET1, increase based on a progressive loading burden by sMR and may thus serve as better predictors of sMR progression.

## 1 INTRODUCTION

Secondary mitral regurgitation (sMR) is highly prevalent among patients with heart failure with reduced ejection fraction (HFrEF) and has a significant impact on morbidity and mortality despite guideline‐directed therapy (GDT).^1^ SMR drives progressive adverse ventricular remodelling, thereby contributing to symptom aggravation and development of advanced heart failure stages.^1^, ^2^ Secondary MR is a complex disease, presenting both, diagnostic and therapeutic challenges. The primary mechanisms of sMR involve mitral annular dilatation and mitral leaflet restriction secondary to left ventricular (LV) remodelling^3^ and a strong dynamic component that fuels HF severity towards irreversible late failure phenotypes.^2^ Apart from aggravation of HF, progression of sMR is associated with even worse prognosis.^2^, ^3^ Treatment approaches such as percutaneous or surgical mitral valve repair (pMVR) or replacement have been attempted in HF patients with severe sMR. Most recent data from the COAPT trial proved safety, improved outcome and increased quality of live by transcatheter mitral leaflet approximation, also focusing treatment initiation in patients with moderate‐to‐severe sMR.^4^ However, considering the contradictory results from the MITRA‐FR trial, beneficial effects by mitral valve repair remain conflicting.^5^, ^6^ Phenotyping an ideal patient population for the correction of sMR and the optimal time‐point for it are fundamental but utterly unknown. Therefore, a more holistic view on pathophysiology is crucial for a profound understanding.

Neurohormones substantially improved our understanding of disease progression in systolic heart failure in general. It is presumed that volume overload on a primary failing ventricle due to a reverse volume shift increases diastolic wall stress and consequently stimulates further modifications at molecular, cellular, tissue and cardiac chamber level including an up‐regulation of systemic and local neurohormonal activation.^7^, ^8^, ^9^, ^10^ Neurohormones, such as N‐terminal pro–B‐type natriuretic peptide (NT‐proBNP), mid‐regional pro‐atrial natriuretic peptide (MR‐proANP), mid‐regional pro‐adrenomedullin (MR‐proADM), C‐terminal pro‐endothelin‐1 (CT‐proET‐1) and copeptin are known to illustrate the hemodynamic and volume state of the cardiovascular system.^3^, ^7^, ^11^, ^12^, ^13^ Elevated levels have been shown to indicate early states of myocardial deterioration in various diseases and have been proven to be excellent markers of outcome in stable chronic systolic heart failure.^2^, ^14^, ^15^, ^16^ More importantly, among assessed markers, some are released from the myocardium, for example natriuretic peptide NT‐proBNP (predominantly produced in the left ventricle) and MR‐proANP (predominantly stored in the atrial tissue and primarily triggered by changes in atrial transmural pressure, in response to volume expansion and atrial stretch) and cover haemodynamics on a cardiac specific level, whilst others are more systemic—rather than cardiac specific—origin (eg MR‐proADM, CT‐proET‐1, copeptin). These peripheral released and acting hormones might represent sMR progression and heart failure not as an organ localized, but a systemically deteriorating condition. Neurohormones have been partly tested in primary MR.^8^ However, despite integrated approaches in the assessment of sMR, the relationship between neurohumoral profiles and longitudinal dynamic of sMR has not been investigated yet, but might help to expand our understanding from morphologic to functional alterations and outcome‐related progression. We therefore sought to assess the predictive power of neurohumoral profiles using a complementary multi‐parametric biomarker set in patients with HFrEF and to explore their ability to predict progression of sMR.

## 2 METHODS

### 2.1 Study population

We enrolled consecutive adult patients with HF with reduced ejection fraction (HFrEF) who presented to our HF clinic at the Vienna General Hospital, a university‐affiliated tertiary centre in this observational, noninterventional study as previously described.^17^ Heart failure with reduced ejection fraction was defined in line with the guidelines as history of HF signs and symptoms as well as a LV ejection fraction (LVEF) below 40%.^18^ As the investigated population already received OMT at index time, there is a portion of patients in New York Heart Association (NYHA) stage I and improved ejection fraction >40%. Baseline examination included medical history, detailed assessment of current medication and an electrocardiogram. Cardiovascular risk factors were recorded according to the respective guidelines as previously described.^1^ All patients underwent a comprehensive echocardiographic examination at our institution at baseline and yearly thereafter within 3 years after study inclusion. SMR progression was defined as advance of ≥one grade in severity with transition to ≥moderate during three years of follow‐up. Analogously, regression of severe sMR was defined as decrease in at least one grade. Exclusion criteria were more than mild aortic or mitral stenosis or moderate primary mitral regurgitation. The primary endpoint was sMR progression compared to baseline echocardiographic examination assessed by a yearly follow‐up. All included patients had to be at least 18 years of age and provided written informed consent to study participation. The study protocol complies with the Declaration of Helsinki and was approved by the ethics committee of the Medical University of Vienna.

### 2.2 Laboratory assessment

Neurohumoral profiles were assessed from venous blood samples drawn from a peripheral vein at index time. Plasma was immediately centrifuged, and samples were frozen at −70°C until assay and analysis were performed. Routinely available laboratory parameters were analysed on‐site, according to the local laboratory's standard procedures. Additional cardiac biomarker N‐terminal B‐type natriuretic peptide (NT‐proBNP) was measured in heparin plasma using the Elecsys Systems (Roche Diagnostics). Mid‐regional pro‐adrenomedullin (MR‐proADM), mid‐regional pro‐atrial natriuretic peptide (MR‐proANP), C‐terminal pro‐endothelin‐1 (CT‐pro‐ET‐1) and copeptin were determined in EDTA plasma using specific sandwich immunoassays (BRAHMS).

### 2.3 Echocardiographic assessment

Baseline and follow‐up echocardiograms were performed at our institution using commercially available equipment (Vivid7, GE Healthcare, and Acuson Sequoia, Siemens). Cardiac morphology was assessed in standard four‐ and two‐chamber views. LV ejection fraction was calculated using the biplane Simpson's method, and semi‐quantitative assessment of right heart function was performed by experienced readers using multiple acoustic windows and graded as normal, mild, mild‐to‐moderate, moderate, moderate‐to‐severe and severe. Mitral regurgitation was quantified by an integrated approach comprising mitral valve morphology, width of the proximal regurgitant jet, proximal flow convergence and pulmonary venous flow pattern.^19^ Valvular stenosis and regurgitation were quantified using an integrated approach and graded as none, mild, mild‐to‐moderate, moderate, moderate‐to‐severe and severe according to the respective guidelines.^19^, ^20^, ^21^ Systolic pulmonary artery pressures (sPAPs) were calculated by adding the peak tricuspid regurgitation (TR) systolic gradient to the estimated central venous pressure.

### 2.4 Statistical analysis

Continuous data were presented as median and interquartile range (IQR) and compared by using the Kruskal‐Wallis test. Discrete data were presented as count and percentage and analysed by using a chi‐square test.

Univariate logistic regression analysis assessing cardiac biomarkers at baseline for sMR progression was applied. To account for potential confounding effects, multivariate logistic regression analysis was performed adjusting for a clinical confounder model including age, gender, kidney function and blood pressure and an echocardiographic confounder model, including changes in LV function and left atrial diameter during follow‐up as well as MR severity at baseline. Results are presented as odds ratios (OR) for a 1‐standard deviation (SD) change in continuous variables with the respective 95% confidence intervals (95% CIs). Kaplan‐Meier analysis was applied to evaluate the effect of sMR progression on survival and compared using log‐rank test. Two‐sided *P*‐values < .05 were used to indicate statistical significance. The SPSS 24.0 software (IBM Corp) was used for all analyses.

## 3 RESULTS

### 3.1 Baseline characteristics

From February 2001 to November 2006, a total of 249 HFrEF patients were prospectively enrolled. Median age was 58 years (IQR: 51‐63), and 208 patients (84%) were male. Aetiology of HF was ischaemic in 90 patients (36%), and sixty‐six per cent of patients (n = 163) were in NYHA class III or IV. Median baseline LV ejection fraction was 26% (21‐35) at index time. Among the 191 (77%) patients with nonsevere sMR at baseline, 34 (14%) showed sMR progression during 3 years of follow‐up. Among patients who remained stable at follow‐up, n = 104 (66%) presented with mild sMR, whereas n = 53 (34%) suffered from at least moderate sMR at baseline. In patients with progression of sMR over time, n = 4 (12%) had mild sMR at baseline, whilst n = 30 (88%) presented with moderate sMR. Regression of sMR occurred in 13 (5%) patients with severe sMR at baseline. At median follow‐up of 61 months (IQR 50‐72), 61 patients died in this study cohort. Mortality was significantly higher among patients with progressive sMR compared to patients with stable sMR (53% vs 27%, *P* = .004). NT‐proBNP was markedly elevated with a median of 2453 pg/mL (IQR:935‐5002). Median (IQR) serum levels of cardiac biomarkers were 299 pmol/L (145‐495) for MR‐proANP, 0.68 nmol/L (0.45‐1.06) for MR‐proADM, 11 pmol/L (6‐22) for copeptin and 62 pmol/L (31‐106) for CT‐pro‐ET1, respectively. Detailed baseline characteristics of the entire study population are displayed in Table 1 *.*

**Table 1:** Baseline characteristics of total study population (n = 249) according to stages of secondary mitral regurgitation

	sMR (total study population) (n = 249)	sMR stable (n = 157)	sMR progression (n = 34)	sMR severe (n = 58)	Overall *P*‐value	Severe vs progressive *P*‐value	Stable vs progressive *P*‐value
Baseline characteristics							
Age, median years (IQR)	58 (51‐63)	58 (51‐63)	58 (51‐64)	58 (50‐64)	.99	.94	.997
Male sex, n (%)	208 (84)	208 (84)	27 (79)	48 (83)	.74	.69	.45
BMI, kg/m^2^ (IQR)	26 (24‐29)	26 (24‐29)	26 (24‐28)	25 (23‐28)	**.009**	.39	.14
Ischaemic aetiology of HF, n (%)	90 (36)	90 (36)	11 (32)	18 (31)	.51	.896	.45
Hypertension, n (%)	118 (47)	118 (47)	16 (47)	18 (31)	**.014**	.126	.50
Diabetes, n (%)	56 (23)	56 (23)	5 (15)	8 (14)	.054	.90	.12
Hypercholesterolaemia, n (%)	152 (61)	152 (61)	10 (29)	16 (28)	**.030**	.85	.09
Left bundle branch block, n (%)	69 (28)	69 (28)	12 (35)	11 (19)	.38	.44	.17
Atrial fibrillation, n (%)	42 (17)	42 (17)	5 (15)	11 (19)	.86	.61	.79
Systolic blood pressure, mmHg(IQR)	115 (100‐130)	115 (100‐130)	110 (7‐120)	100 (90‐117)	**<.001**	**.033**	**.052**
Diastolic blood pressure, mmHg(IQR)	70 (60‐80)	70 (60‐80)	70 (60‐80)	65 (60‐75)	**<.001**	.31	**.007**
Heart rate, beats per minute(IQR)	72 (62‐82)	72 (62‐82)	72 (62‐81)	71 (65‐90)	.13	.28	.82
NYHA functional class					**<.001**	.44	**.003**
NYHA I, n (%)	19 (8)	14 (9)	1 (3)	4 (7)			
NYHA II, n (%)	67 (27)	53 (34)	8 (24)	6 (10)			
NYHA III, n (%)	112 (45)	75 (48)	13 (38)	24 (41)			
NYHA IV, n (%)	51 (21)	15 (10)	12 (35)	24 (41)			
Creatinine, mg/dL (IQR)	1.2 (1.0‐1.4)	1.1 (1.0‐1.3)	1.2 (1.1‐1.3)	1.3 (1.1‐1.5)	**.008**	.41	.09
Blood urea nitrogen, mg/dL(IQR)	20 (17‐30)	20 (16‐27)	21 (18‐33)	27 (20‐38)	**<.001**	.22	.09
Echocardiographic baseline examination							
Left ventricular end‐diastolic diameter, mm (IQR)	64 (58‐70)	62 (55‐68)	65 (60‐70)	68 (62‐75)	**<.001**	**.034**	.09
Left ventricular function							
Moderately reduced (EF 30%‐40%), n (%)	71 (29)	54 (34)	9 (26)	8 (14)	**.012**	.13	.37
Severely reduced (EF < 30%), n (%)	141 (57)	71 (45)	21 (62)	49 (85)	**<.001**	**.014**	.08
Left atrial diameter, mm (IQR)	65 (58‐72)	61 (55‐69)	65 (62‐73)	72 (68‐77)	**<.001**	**.001**	**.004**
Right atrial diameter, mm (IQR)	58 (52‐66)	56 (51‐64)	58 (52‐68)	65 (59‐73)	**<.001**	**.007**	.30
Right ventricular end‐diastolic diameter, mm (IQR)	36 (32‐41)	35 (31‐38)	37 (33‐41)	41 (35‐47)	**<.001**	**.011**	.07
Systolic pulmonary artery pressure, mmHg (IQR)	48 (39‐56)	43 (36‐50)	47 (37‐57)	56 (48‐64)	**<.001**	**.025**	.31
Mitral regurgitation:					**<.001**	**<.001**	**<.001**
≤mild, n (%)	108 (43)	104 (66)	4 (12)	0 (0)			
≥moderate, n (%)	141 (57)	53 (34)	30 (88)	58 (100)			
Tricuspid regurgitation (≥ moderate), n (%)	58 (23)	14 (9)	8 (24)	36 (62)	**<.001**	**<.001**	**.016**
Neurohormones							
NT‐proBNP, pg/mL (IQR)	2453 (935‐5002)	1784 (664‐3615)	2740 (1258‐5457)	4212 (2588‐8112)	**<.001**	**.023**	**.031**
MR‐proANP, pmol/L (IQR)	299 (145‐495)	205 (108‐371)	314 (188‐485)	524 (368‐789)	**<.001**	**.001**	**.009**
MR‐proADM, nmol/L (IQR)	0.68 (0.45‐1.06)	0.60 (0.41‐0.85)	0.78 (0.56‐1.15)	1.00 (0.54‐1.71)	**<.001**	.17	**.004**
Copeptin, pmol/L (IQR)	11 (6‐22)	9 (5‐17)	10 (5‐29)	21 (11‐38)	**<.001**	**.010**	.38
CT‐pro‐ET1, pmol/L (IQR)	62 (31‐106)	52 (27‐91)	71 (45‐142)	95 (38‐161)	**.001**	.59	**.011**
Guideline‐directed heart failure therapy							
RAS antagonist, n (%)	242 (97)	154 (98)	32 (94)	56 (97)	.42	.58	.19
Beta‐blockers, n (%)	189 (76)	129 (82)	30 (88)	30 (52)	**<.001**	**<.001**	.39
Mineralocorticoid antagonist, n (%)	90 (36)	55 (35)	15 (44)	20 (35)	.58	.36	.32
Furosemide, n (%)	194 (78)	112 (71)	28 (82)	54 (93)	**.002**	.11	.19
Cardiac resynchronization therapy, n (%)	43 (21)	25 (20)	10 (36)	8 (14)	.07	**.022**	.08

Note

Values are median (interquartile range (IQR)) or n (%).

Bold values are statistically significant.

Abbreviations: BMI, body mass index; CT‐ET‐1, C‐terminal pro‐endothelin‐1; MR‐proADM, mid‐regional pro‐adrenomedullin; MR‐proANP, mid‐regional pro‐atrial natriuretic peptide; NT‐proBNP, N‐terminal pro–B‐type natriuretic peptide; RAS, renin‐angiotensin system; sMR, secondary mitral regurgitation.

### 3.2 Progression of secondary mitral regurgitation

Of 191 patients with nonsevere sMR at baseline 157 (82%) remained stable, whereas 34 (18%) showed progressive sMR within 3 years after study enrolment. Those patients experiencing progression of sMR were more symptomatic at baseline in contrast to patients with stable sMR (NYHA class IV 35% vs 10%; *P* = .003), but comparable to symptomatic status of those who presented with severe sMR at baseline (*P* = .44). On baseline echocardiographic examination, the occurrence of progressive sMR was associated with larger left atrial size (*P* = .004) and more severe tricuspid regurgitation compared to stable sMR (*P* = .02), but significantly less than in patients with severe sMR (Table 1).

### 3.3 Neurohumoral activation in progressive secondary mitral regurgitation

**Figure 1:**
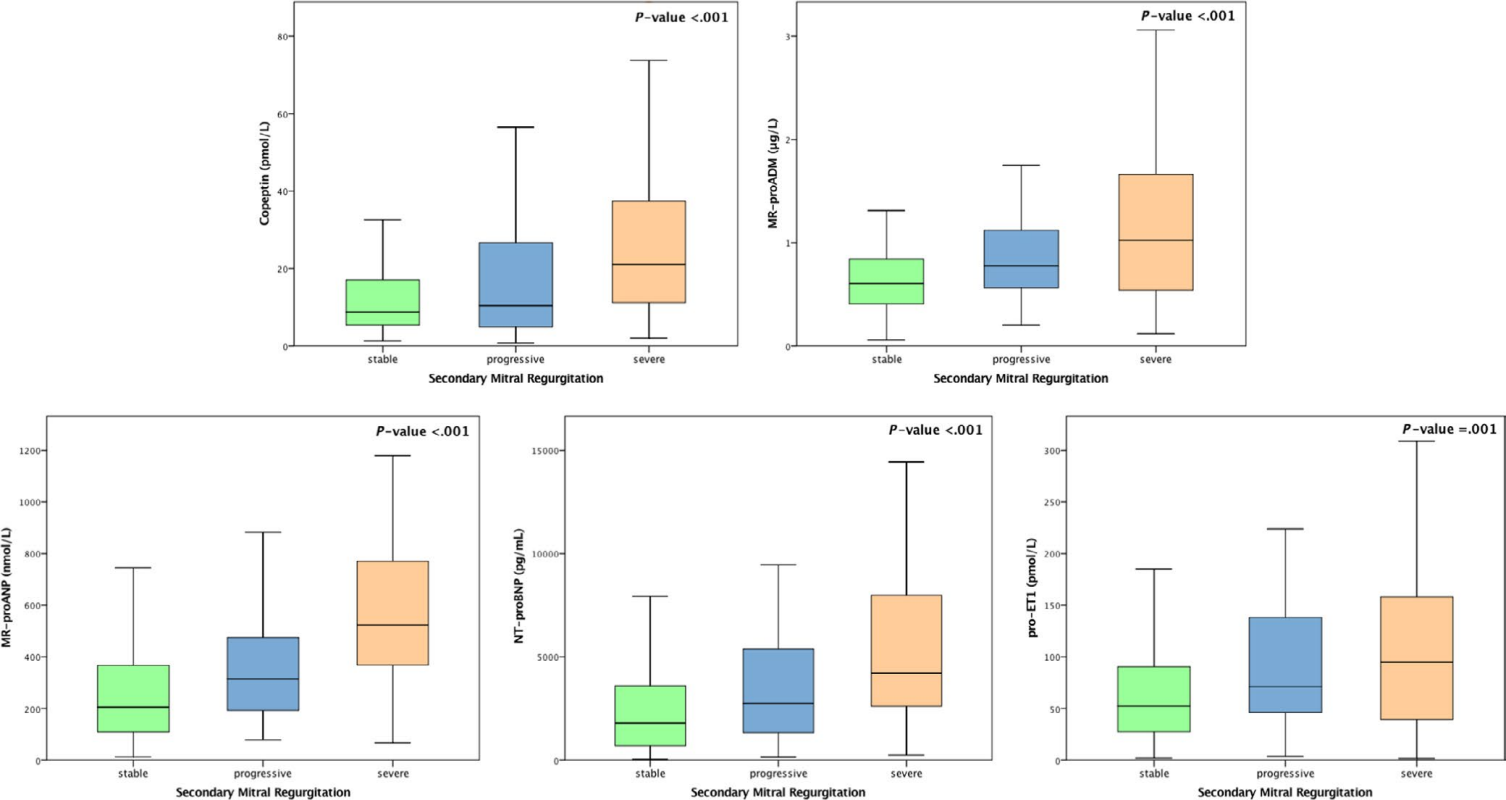
Neurohumoral activation in different stages of secondary mitral regurgitation. Levels are displayed as Tukey boxplots. Group comparisons between stable vs progressive vs severe sMR were made by the Kruskal‐Wallis test

Neurohumoral profiles were elevated in all patients with a sequential activation according to sMR dynamics defined as nonsevere, stable, progressive and severe (NT‐proBNP; MR‐proANP; MR‐proADM; copeptin: *P* < .001, for all; CT‐pro‐ET1 *P* = .001) (Figure 1).

In subgroup analyses comparing subsequent progression of sMR with stable on the one hand and severe on the other, values of neurohumoral activation in progressive sMR differed significantly for NT‐proBNP (progressive vs severe: 2740 pg/mL (1258‐5457 [IQR]) vs 4212 pg/mL (2588‐8112 [IQR]); *P* = .023; progressive vs stable: 2740 pg/mL (1258‐5457 [IQR]) vs 1784 pg/mL (664‐3615 [IQR]); *P* = .031) and MR‐proANP (progressive vs severe: 314 pmol/L (188‐485 [IQR]) vs 524 pmol/L (368‐789 [IQR]); *P* = .001; progressive vs stable: 314 pmol/L (188‐485 [IQR]) vs 205 pmol/L (108‐371 [IQR]); *P* = .009). Activation of MR‐proADM and CT‐proET‐1 was significantly increased only in progressive sMR compared to stable patients (0.78 nnmol/L (0.56‐1.15 [IQR]) vs 0.60 nnmol/L (0.41‐0.85 [IQR]); *P* = .004; 71 nnmol/L (45‐142 [IQR]) vs 52 nnmol/L (27‐91 [IQR]); *P* = .011, respectively), without differences between progressive and severe sMR. Copeptin was highly overactivated in severe compared to progressive sMR (21 pmol/L (11‐38 [IQR]) vs 10 pmol/L (5‐29 [IQR]); *P* = .010) (Table 1), but did not differ between stable and progressive sMR.

**Figure 2:**
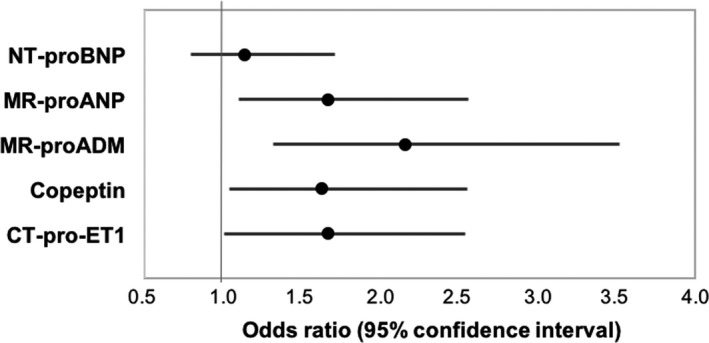
Results of the logistic regression analysis illustrating the association of biomarkers with risk for sMR progression. Forest plot displaying the association of the neurohumoral biomarkers and progression of sMR. Odds ratios (OR) refer to a 1‐SD increase/decrease in continuous variables

Baseline MR‐proADM (OR 2.16, 95% CI 1.32‐3.52; *P* = .002), MR‐proANP (OR 1.67, 95% CI 1.10‐2.56; *P* = .017), copeptin (OR 1.63, 95% CI 1.04‐2.55; *P* = .03) and CT‐pro‐ET1 (OR 1.67, 95% CI 1.01‐2.54; *P* = .016) predicted sMR progression during 3 years of follow‐up. Contrarily, the gold standard cardiac biomarker NT‐proBNP was not significantly associated with sMR progression (OR 1.14, 95% CI 0.80‐1.71; *P* = .52). Forest plot displaying the univariate logistic regression results of the respective cardiac biomarkers for sMR progression is depicted in Figure 2. Neurohumoral biomarker association remained significant after adjustment for age, gender, kidney function and blood pressure [adjusted OR for MR‐proADM (adj. OR 2.25, 95% CI 1.29‐3.93; *P* = .004), MR‐proANP (adj. OR 1.84, 95% CI 1.14‐3.00; *P* = .012), copeptin (adj. OR 1.66, 95% CI 1.04‐2.67; *P* = .04) and CT‐pro‐ET1 (adj. OR 1.68, 95% CI 1.06‐2.68; *P* = .027)]. NT‐proBNP was not associated with sMR progression (adj. OR 1.16, 95% CI 0.72‐1.89; *P* = .54). Furthermore, results remained virtually unchanged after adjustment for our echocardiographic confounder model (Table 3). Detailed results of the logistic regression analyses are presented in Tables 2 and 3.

**Table 2:** Univariate logistic regression analysis assessing risk factors for sMR progression

	SD	OR	95% CI	*P*‐value	ROC
Neurohormones					
NT‐proBNP	5819	1.14	0.80‐1.71	.52	0.62
MR‐proANP	366	1.67	1.10‐2.56	**.017**	0.64
MR‐proADM	0.76	2.16	1.32‐3.52	**.002**	0.67
Copeptin	25	1.63	1.04‐2.55	**.034**	0.55
CT‐pro‐ET1	69	1.67	1.01‐2.54	**.016**	0.64

Note

Odds ratios (OR) refer to a 1‐SD (standard deviation) increase in continuous variables. Bold values indicate statistical significance.

Abbreviations: CT‐ET‐1, C‐terminal pro‐endothelin‐1; MR‐proADM, mid‐regional pro‐adrenomedullin; MR‐proANP, mid‐regional pro‐atrial natriuretic peptide; NT‐proBNP, N‐terminal pro–B‐type natriuretic peptide; RAS, renin‐angiotensin system.

**Table 3:** Multivariate logistic regression analysis assessing risk factors for sMR progression

	SD	Clinical confounder model^a^			Echocardiographic confounder model^b^		
		OR	95% CI	*P*‐value	OR	95% CI	*P*‐value
Neurohormones							
NT‐proBNP	5819	1.16	0.72‐1.89	.54	1.24	0.80‐1.93	.33
MR‐proANP	366	1.84	1.14‐3.00	**.012**	1.85	1.16‐2.94	**.010**
MR‐proADM	0.76	2.25	1.29‐3.93	**.004**	2.85	1.56‐5.22	**.001**
Copeptin	25	1.66	1.04‐2.67	**.035**	2.59	1.44‐4.67	**.002**
CT‐pro‐ET1	69	1.68	1.06‐2.68	**.027**	1.87	1.18‐2.98	**.008**

Note

Odds ratios (OR) refer to a 1‐SD (standard deviation) increase in continuous variables.

Bold values indicate statistical significance.

a Adjusted for age, gender, kidney function, blood pressure.

b Adjusted for changes in LV function and left atrial diameter at follow‐up and MR severity at baseline.

### 3.4 Neurohumoral activation and regression of severe secondary mitral regurgitation

Among patients with severe sMR at baseline, regression of sMR occurred in 13 patients (22%). Secondary MR regression was not associated with neurohumoral improvement of neurohumoral profiles in the logistic regression model (all *P* > .5; detailed data not shown). Due to limited numbers, results need to be interpreted with caution.

## 4 DISCUSSION

The present long‐term observational study introduces biomarker profiling as a potent tool to identify patients at risk for sMR progression despite GDT. Specifically, MR‐proANP, copeptin, CT‐pro‐ET1 and MR‐proADM are powerful predictors of sMR progression even after adjusting of clinical confounders. In contrast, the usefulness of the gold standard biomarker NT‐proBNP is limited in predicting sMR progression.

### 4.1 Progression of secondary mitral regurgitation—Who is at risk?

An increasing body of evidence consolidates the impact of sMR on the failing LV and long‐term outcome even in patients under GDT.^1^ Importantly, recent data illustrate the progressive nature of sMR to be a key feature that drives mortality in HFrEF patients.^2^ In fact, progressive sMR occurs in 20%‐30% of patients with nonsevere sMR despite GDT and is independently associated with a more than twofold increased risk of death.^1^ Defining patients at risk for progression is mandatory as in this patient subpopulation more invasive early treatment strategies are available but the level of evidence in guideline recommendations is weak.^22^ Whilst it has been shown that invasive treatment for sMR can delay or even prevent progressive HF, characteristics of patients that may benefit most and the optimal time‐point for intervention are not ultimately determined.^4^, ^23^ Neurohumoral serum markers may represent a valuable addition in this respect and might help to identify patients with nonsevere sMR at high risk of impeding progression, who would potentially benefit from early referral to a specialized heart valve centre, thus potentially not delaying important interventions.

### 4.2 Neurohumoral profiling in secondary mitral regurgitation

The cardiovascular hormones NT‐proBNP, BNP, MR‐proANP, MR‐proADM, copeptin and endothelin‐1 have never been described regarding their prognostic impact on sMR progression in HFrEF. In the present study population under GDT, all assessed biomarkers were elevated^24^, ^25^, ^26^, ^27^, ^28^ with increasing severity of sMR (Table 1). Among those patients with nonsevere sMR at baseline, determination of all biomarkers with exception of copeptin helped to identify patients with subsequent sMR progression, who displayed increased circulating neurohormone levels disparate from patients with stable sMR. Patients with severe sMR had a further significant increase in NT‐proBNP, MR‐proANP and copeptin compared to those who progressed, but MR‐proADM and CT‐pro‐ET1 were comparable. After unadjusted as well as adjusted regression analysis, NT‐proBNP lost its significance to predict sMR progression, whilst all other hormones remained significantly associated.

### 4.3 Natriuretic peptides and sMR progression

MR‐proANP was the only marker with significantly different baseline values between all analysed subgroups (stable sMR vs subsequent sMR progression vs severe sMR) and positively associated with risk of progression. MR‐proANP is released from the myocardium, but contrarily to NT‐proBNP predominantly stored in the atrial tissue.^29^ Its production is primarily triggered by changes in atrial transmural pressure, in response to volume expansion and atrial stretch, common pathophysiologic incidents in sMR.^30^ Apparently enlarged atria and increased levels of MR‐proANP mirror the functional burden of the cardiac system already before morphologic changes in the mitral valve are trackable and therapeutic interventions are potentially beneficial.

Interestingly, our data proved that levels of NT‐proBNP were disparate among groups of sMR: stable, progressive, severe, likely to reflect the enhanced hemodynamic burden on the left ventricle in HFrEF. However, NT‐proBNP was not useful to predict progression of sMR in the present regression analysis. As only a minor fraction of NT‐proBNP is produced by the left atrium and plenty by the LV, a potential solution might be a volume shift directed towards the left atrium related to increasing atrial pressure and a systolic unloading of the left ventricle. Deductively, this pressure alteration might explain why NT‐proBNP does not predict sMR progression compared to the predictive value of MR‐proANP.

### 4.4 Adrenomedullin and sMR progression

A further marker of interest with a more peripheral rather than cardiac origin is MR‐proADM.^31^ In our observation, MR‐proADM was significantly altered between stages of nonsevere sMR, diverging and predicting patients at risk for sMR progression from stable nonsignificant sMR. In univariate and multivariate logistic regression analyses, MR‐proADM represents a robust predictor of progression. Classifying MR‐proADM as a marker of forward failure, our findings reflect a difference in the systemic alteration in patients at risk for sMR progression compared to those who remain stable and underline the systemic beyond the cardiac burden in this subset of patients.

### 4.5 Regression of secondary mitral regurgitation

Guideline‐directed therapy has been found to improve sMR severity.^5^ Effects by medical therapy alone have been linked to the beneficial effects of the neurohumoral system on LV remodelling.^32^ Further, clinically meaningful symptomatic improvement by cardiac resynchronization therapy (CRT) has been associated with a decrease in the effective regurgitant orifice and regurgitant volumes.^33^ In our observation, a subset of patients under GDT also experienced regression of sMR. Neurohumoral levels at baseline could not predict sMR regression. However, based on a small sample size, results need to be interpreted with caution. As in this study cohort regression of mitral regurgitation was not attributable to additional therapeutic interventions, the distinct underlying mechanisms have to be further investigated. However, invasive therapeutic strategies to reduce sMR and to improve outcome exist but data on the net effect on outcome remain controversial regarding the optimal timing of intervention.^4^, ^6^ At this point, we need to further question the ‘if’ and ‘when’ regarding the optimal timing for possibly more invasive therapeutic strategies to disrupt the vicious cycle of progressive secondary mitral regurgitation in heart failure at an early stage.

## 5 LIMITATION

The study reflects the experience of a single tertiary care centre. However, this ensures the inclusion of a homogenous patient population, a consistent quality of imaging procedures and blood sample processing as well as adherence to a consistent clinical routine. As there is no uniform definition of progressive MR, we defined MR progression as advance of at least one degree in severity according to the respective guidelines.^13^, ^19^ A definition of MR progression based on quantitative measures of MR severity would be of potential interest; however, this would mean to have a distinct definition of progression for each respective parameter, which makes overall assessment complex and significance in clinical practice limited. Therefore, this study focused on aiming for a more global model characterizing the probability of sMR progression, helping to understand pathophysiologic processes in the course of sMR progression and deterioration of heart failure.

## 6 CONCLUSION

Increased plasma levels of neurohumoral cardiac biomarkers are predictors of sMR progression, suggesting a potential role to guide clinical workup and follow‐up in patients with HFrEF. NT‐proBNP was not useful to predict progressive MR in the present analysis. The exact mechanism behind this finding might be related to a relative unloading of the LV in the presence of MR, leading to a volume shift directed towards the left atrium. MR‐proANP, primarily produced in the atria, copeptin partly triggered by intra‐cardiac and intra‐arterial pressures and MR‐proADM, a marker of forward failure and peripheral released vasoactive CT‐proET1, reflect systemic alterations in patients at risk for sMR progression. Their increase follows a progressive loading burden induced by sMR which makes them being robust predictors of sMR progression.

## CONFLICT OF INTEREST

The authors declare no conflict of interest.

## AUTHOR CONTRIBUTIONS

GG, HA and MH conceptualize the data; GG, HA and MH contributed to methodology; GG, HA, PE.B. and MH involved in validation; GG and HA contributed to formal analysis; HA, GG, PE.B., NP and GH investigated the study data; MH, GG and CH provided resources; GG, HA and PE.B. curated the data; HA wrote—original draft preparation; GG, PE.B., NP, G.H, JM, CH and MH wrote—review and editing; HA visualized the study; GG and MH supervised the study; GG and MH administrated the project.
